# DYADIC INTERVENTION FOR PEOPLE WITH DEMENTIA AND THEIR FAMILY CARERS AFTER THE DIAGNOSIS

**DOI:** 10.1093/geroni/igz038.411

**Published:** 2019-11-08

**Authors:** Marleen Prins, Marjolein Veerbeek, Claudia van der Velden, Bernadette Willemse

**Affiliations:** 1 Netherlands Institute of Mental Health Addiction (Trimbos-institute), Utrecht, Utrecht, Netherlands; 2 Trimbos-institute, Netherlands

## Abstract

Adaptation to and coping with the diagnosis dementia is a complex process. The right support in this phase is important to find the right care and to (self-)manage future care wishes. However, people with dementia (PWD) and their family carers (FC) often experience support directly after the diagnosis as inadequate. Support is not provided until problems start to accumulate and available support is not focused on continuation of their life and the emotional impact of the diagnosis. Therefore, the intervention SHARE [Support, Health, Activities, Resources, Education] was developed and studied in the US and adjusted to and pilot-tested in the Netherlands with positive results. SHARE is innovative because it is designed for dyads dealing with early-stage dementia to enhance communication between PWD and their FC and to prepare them for the future. In this study, the Dutch SHARE intervention was adjusted because of new insights and an RCT is being conducted to evaluate the (cost)effectiveness of this version of the Dutch SHARE. The primary outcome measurement for the PWD is quality of life and self-efficacy for the FC. Secondary outcomes are stress, communication in the relationship and perspective taking (only FC). The design and procedures of the RCT will be presented in this poster session as well as the demographic characteristics of participating dyads. This study contributes to knowledge about psychosocial interventions for PWD and FC with special attention for preventive empowerment of the ability to cope with the disease and the capacity to deal with the situation.

